# 422 Million intrinsic quality factor planar integrated all-waveguide resonator with sub-MHz linewidth

**DOI:** 10.1038/s41467-021-21205-4

**Published:** 2021-02-10

**Authors:** Matthew W. Puckett, Kaikai Liu, Nitesh Chauhan, Qiancheng Zhao, Naijun Jin, Haotian Cheng, Jianfeng Wu, Ryan O. Behunin, Peter T. Rakich, Karl D. Nelson, Daniel J. Blumenthal

**Affiliations:** 1 Honeywell International, Phoenix, AZ USA; 2 Department of Electrical and Computer Engineering, University of California Santa Barbara, Santa Barbara, CA USA; 3 Department of Applied Physics, Yale University, New Haven, CT USA; 4 Department of Physics and Astronomy, Northern Arizona University, Flagstaff, AZ USA; 5 Center for Materials Interfaces in Research and Applications, Northern Arizona University, Flagstaff, AZ USA

**Keywords:** Integrated optics, Optical materials and structures

## Abstract

High quality-factor (Q) optical resonators are a key component for ultra-narrow linewidth lasers, frequency stabilization, precision spectroscopy and quantum applications. Integration in a photonic waveguide platform is key to reducing cost, size, power and sensitivity to environmental disturbances. However, to date, the Q of all-waveguide resonators has been relegated to below 260 Million. Here, we report a Si_3_N_4_ resonator with 422 Million intrinsic and 3.4 Billion absorption-limited Qs. The resonator has 453 kHz intrinsic, 906 kHz loaded, and 57 kHz absorption-limited linewidths and the corresponding 0.060 dB m^−1^ loss is the lowest reported to date for waveguides with deposited oxide upper cladding. These results are achieved through a careful reduction of scattering and absorption losses that we simulate, quantify and correlate to measurements. This advancement in waveguide resonator technology paves the way to all-waveguide Billion Q cavities for applications including nonlinear optics, atomic clocks, quantum photonics and high-capacity fiber communications.

## Abstract (web summary)

Integrated photonic all-waveguide resonators are a critical component in many future applications. Here the authors develop an optimized photonic all-waveguide resonator with an ultra-high quality factor, Q, of almost half a billion, and a narrow sub-MHz linewidth.

## Introduction

Ultra-high quality-factor (Q) resonators play a critical role across a wide range of applications including ultra-narrow linewidth lasers^1–3^, optical frequency combs^4–6^, optical gyroscopes^7^, optical atomic clocks^8^ and quantum communications and computation^9–13^. Resonators used for laser linewidth narrowing and phase noise reduction have been relegated to benchtop and bulk-optic implementations. Linewidth narrowing is achieved using ultra-high Q resonators (UHQR) in combination with optoelectronic feedback to suppress close-to-carrier frequency noise. Carrier stabilization is handled through temperature stabilization, athermalization, and environmental isolation. A record low 40 mHz laser linewidth with frequency stabilization of 1 × 10^−16^ over 1 s utilized a cryogenically cooled and environmentally isolated silicon Fabry-Perot resonator^1^. Ultra-low expansion glass cavities can realize sub-Hz linewidth semiconductor lasers with stabilization on the order of 2.7 × 10^−15^ over 1 s^14^. Progress has been made to miniaturize these cavities using tapered-fiber and free-space coupled bulk optical resonators^15–20^ to achieve Qs of 63 Billion^18^. Compact, centimeter-scale, microrod cavities with 1 Billion Q have been used to reduce a semiconductor laser integral linewidth to 25 Hz with a 7 × 10^−13^ fractional frequency stability at 20 ms^3^.

Translating the performance of bulk-optic resonators to integrated waveguide designs will lead to a dramatic reduction in size, power, cost, and sensitivity to environmental disturbances as well as enable a higher level of integration^21–23^. Designs with a large mode area and volume can mitigate optical nonlinearities and thermo-optic frequency noise^24,25^. To-date, integrated resonators have been limited to intrinsic *Q* values below 260 Million for ring-based^26^ and 150 Million for spiral-based^25^ waveguide designs. Significant progress has been made with hybrid designs that employ an on-chip etched silica disk resonator, demonstrating 206 Million^27^ and recently a record 1.1 Billion^28^. However, these designs are not fully compatible with wafer-scale fabrication, are susceptible to environmental conditions, and need hermetic sealing as well as careful mode engineering. A significant advance in integrated resonators will directly impact applications that require ultra-low laser linewidth and phase noise, and narrow linewidth filtering will further benefit from structures that can be integrated on chip with other components (e.g., lasers, filters). The challenges involve reducing scattering losses and absorption. Scattering losses due to waveguide imperfections can be reduced through high-aspect-ratio designs^29,30^ and improved lithography and etching^31^, while absorption losses can be reduced by a proper choice of material bandgap^32^, annealing of impurities^22,33^, and passivation of absorptive surface states^34^. If waveguide sidewall and surface roughness are eliminated, the fundamental loss-limited performance will be determined by fundamental material losses and Rayleigh scattering^35–38^. A measure of the waveguide absorption limit indicates the achievable performance for a given waveguide technology, if the scattering mechanisms other than Rayleigh are reduced below the absorption loss. The eventual loss limits will be due to Rayleigh scattering for waveguide materials with the inherent disorder and a residual imaginary part of the refractive index even far away from resonances^31^. Intrinsic loss sets the lower bound for the resonator linewidth. Solutions are needed for all-waveguide resonator designs with Qs approaching 500 Million, capable of exceeding a Billion, with sub-MHz resonances, large mode area and volume, and compatibility with photonic integration and wafer-scale processing.

In this paper, we report a significant advancement in integrated waveguide resonator performance. A Si_3_N_4_ bus-coupled ring-resonator with a measured intrinsic *Q* of 422 Million is demonstrated. The resonator has a 906 kHz full-width half maximum (FWHM) linewidth and a corresponding finesse of 3005, demonstrating the lowest linewidth, to the best of our knowledge, in a photonic integrated planar circuit. We measure a 453 kHz intrinsic linewidth, a 57 kHz loss-limited linewidth, and a corresponding linear loss of 0.060 dB m^−1^, representing, to the best of our knowledge, the lowest waveguide loss on-chip achieved with a deposited SiO_2_ upper cladding^30^. Moreover, we report a 3.4 Billion absorption loss-limited Q measured using a photothermal measurement technique^38,39^. These are the highest intrinsic and absorption loss-limited quality factors and lowest linewidth reported to date for a photonic integrated resonator, to the best of our knowledge. This performance is achieved through a careful reduction of scattering and absorption loss components and the introduction of a thin, ~5 nm, blanket nitride layer and extra anneal step. By modeling scattering loss and measuring total intrinsic loss and absorption loss, we quantify the loss contribution from each loss origin. In addition to exploring these commonly known loss origins, we perform secondary ion mass spectroscopy (SIMS) measurements to investigate the potential dangling bond resonances at the low-pressure chemical vapor deposition (LPCVD) nitride waveguide core surface with and without the ~5 nm blanket nitride layer. We find that with the ~5 nm blanket layer and the 30-min anneal, the hydrogen concentration is reduced by one order of magnitude at the top and bottom of the LPCVD deposited waveguide core. The large resonator mode area and mode volume of this design enable the reduction of low-frequency noise components and the contributions to the integral linewidth originating from thermal dampening of photothermal noise. These results demonstrate the potential to bring the performance of bulk optic and etched resonators to planar all-waveguide solutions and pave the path towards integrated all-waveguide Billion Q cavities for atomic clocks, quantum computing and communications, precision spectroscopy, and energy-efficient coherent communications systems.

## Results

### Resonator design and fabrication

**Fig. 1:**
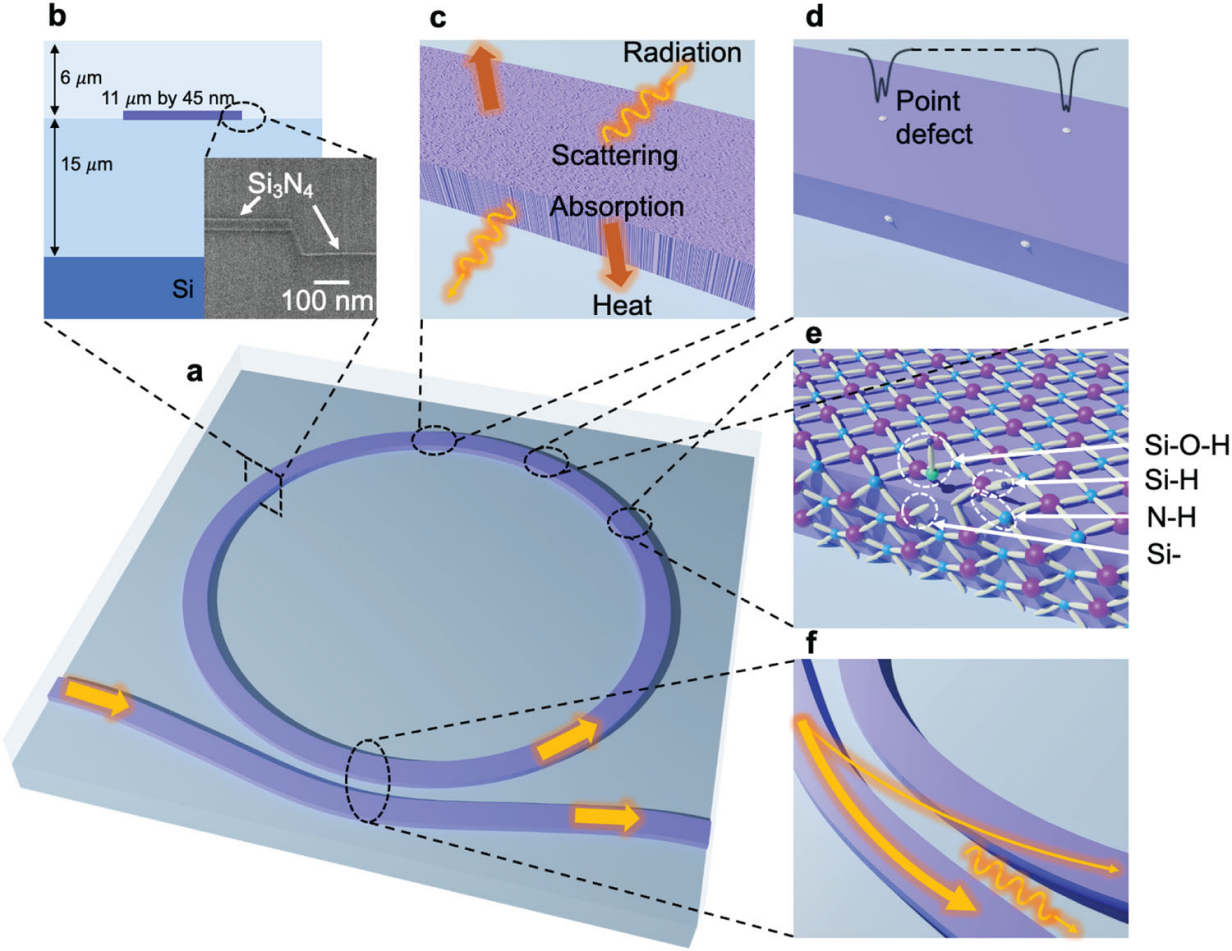
Bus-coupled ring resonator and loss mechanisms. **a** Illustration of the resonator design. **b** Waveguide design. Inset scanning electron micrograph (SEM) image of the waveguide cross section with the thermal oxide bottom cladding, an etched low-pressure chemical vapor deposition (LPCVD) grown silicon nitride waveguide core, an additional thin (~5 nm) blanket layer of silicon nitride (covering the core top and sidewalls), and a tetraethoxysilane pre-cursor plasma-enhanced chemical vapor deposition (TEOS-PECVD) oxide top cladding. SEM length scale bar is 100 nm. **c** Waveguide surface roughness scatters the guide mode energy into radiation mode and bulk absorption generates heat. **d** Point defects split resonances. **e** Defect bonds such as Si–O–H, Si–H, N–H, and dangling bonds lead to surface absorption. **f** The bus-ring coupler scatters energy into radiation modes and adds excess loss.

Our ultra-high Q waveguide ring resonator is illustrated in Fig. 1a, and a scanning electron microscopy (SEM) micrograph cross section is shown in Fig. 1b. Waveguide surface roughness couples guided energy into the radiation continuum resulting in scattering loss, and the bulk material absorption converts the guided modes into heat as illustrated in Fig. 1c. Point defects on the waveguide surface, created during the material deposition or waveguide etching processes, can introduce coupling between longitudinal modes or the forward and backward propagating modes, causing random resonance splitting as illustrated in Fig. 1d. Material deposition and waveguide etching processes can create reconstructed Si–Si bonds and dangling Si- and N- bonds, which can also become secondary bonds with hydrogen impurities such as Si–H, N–H, and Si–O–H, leading to surface absorption loss^38,40^. Bus-to-resonator coupling can introduce resonator loss in the ultra-high Q regime. Careful design of bus-to-resonator coupling is less susceptible to excess loss^26^.

The Si_3_N_4_ resonator waveguide core is a high-aspect ratio, 11-μm wide by 40-nm thick design, chosen to mitigate sidewall scattering losses^26,29^. The bus waveguide core is also a high-aspect ratio, 7-μm wide by 40-nm thick design, that ensures single-mode operation^29^. The final waveguide structure, described in more details below and in the Supplementary Information, contains a ~5 nm blanket nitride layer that covers the waveguide top- and side-walls, and is used to lower the losses and increase the Q. Waveguide mode simulations show that the resonator waveguides support only the fundamental TE mode. The resonator radius, 11.787 mm, is larger than the critical bending radius for the fundamental TE mode, while higher-order modes are not supported due to large bending losses in the asymmetric upper and lower oxide cladding geometry (see Supplementary Fig. 2). The directional coupler is weakly tapered to avoid excess coupler loss^26^. Based on our coupling simulations and prior test device measurements, the 6.898-μm gap between the bus and resonator waveguide realizes an under-coupled resonator design (discussed in more detail in the Supplementary section and Supplementary Fig. 3).

The fabrication process flow follows our prior wafer patterning, etching, upper cladding deposition, and annealing steps^2^ with an additional thin (~5 nm) nitride blanket layer located under the final upper cladding oxide. It is this thin nitride blanket layer that is used to smooth the waveguide top- and side-walls, reducing core surface scattering and compensating for dangling bonds that we believe are created during primary waveguide core LPCVD nitride deposition and nitride waveguide core etch. As described in more detail in the Methods section, a 15 μm thick bottom oxide cladding layer is thermally grown on a silicon substrate. A 40 nm silicon nitride is then deposited by LPCVD and all waveguide cores are etched as described in the Methods section. The nitride core is fully etched and the lower cladding is over-etched, creating a mesa-type core waveguide structure as seen in Fig. 1b. A 5 nm thick LPCVD nitride layer blankets the waveguide top- and side-walls and cladding regions as seen in the SEM photo in Fig. 1b. The increased waveguide width of ~10 nm and ~5 nm nitride layer thickness, and presence of the ~5 nm nitride layer across cladding, does not affect the mode shape, nor alter the desired single-mode operation, as discussed in more detail in the Supplementary Information. Annealing is performed before and after the upper cladding tetraethoxysilane pre-cursor plasma-enhanced chemical vapor (TEOS-PECVD) deposition. We fabricated UHQR devices with the ~5 nm nitride blanket layer and “control” devices without the blanket layer, both with the same bus waveguide and resonator design parameters. As shown in the Supplementary Information, a 5 nm change in the thickness of the waveguide only results in a 0.1% change in the mode effective index, and a 10-nm width change only results in a<0.01% change in the mode effective index.

### Q-factor, linewidth, and resonance splitting

**Fig. 2:**
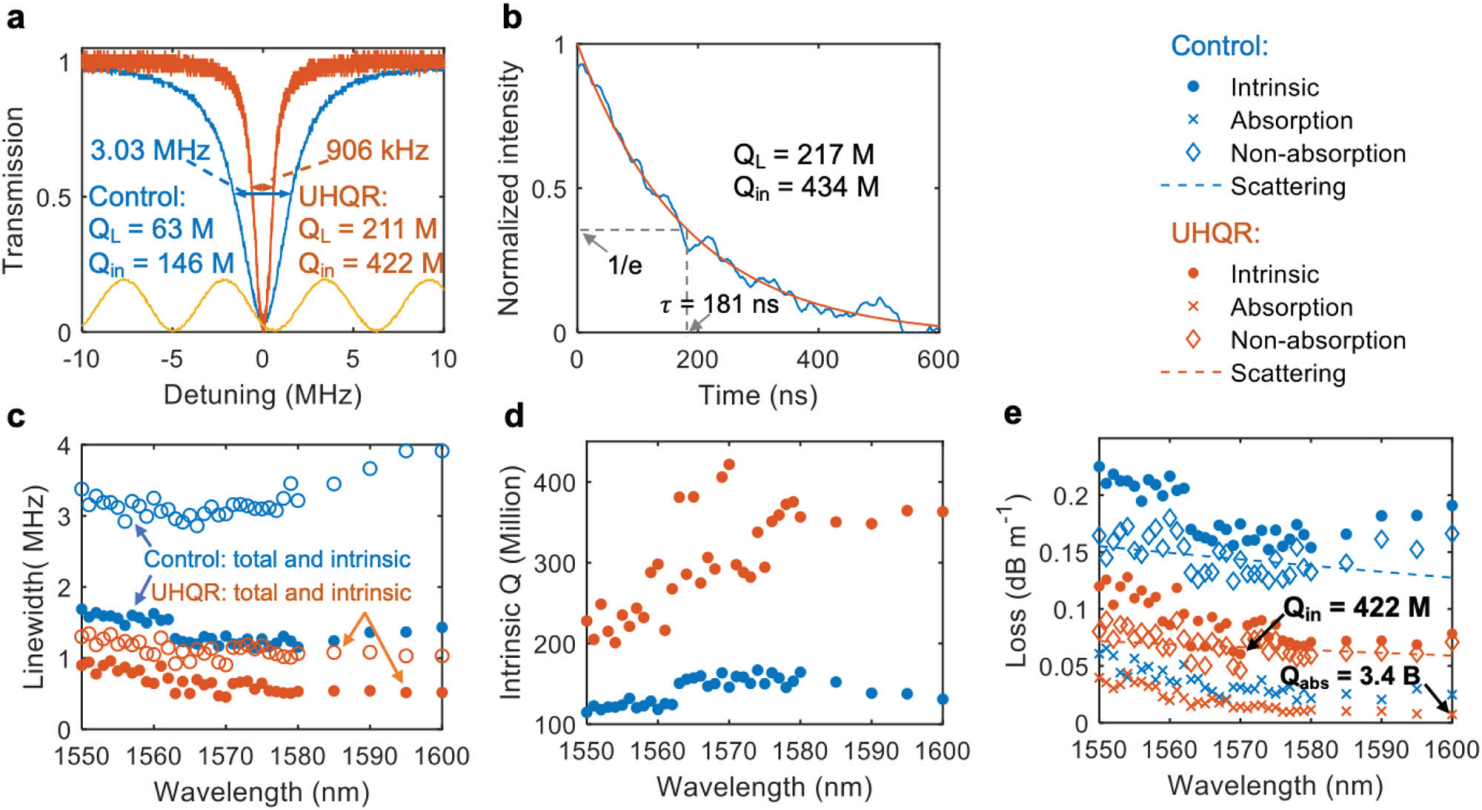
Linewidth, ring-down, and photo-thermal absorption loss measurements of the UHQR and control devices. **a** Spectral scan of the fundamental 1570 nm transverse electric field (TE) mode for the control (blue curve) and ultra-high quality factor (*Q*) resonator (UHQR) (orange curve) devices. The total and intrinsic linewidths are extracted from a Lorentzian fit. The yellow sinusoidal signal is a radio frequency (RF) calibrated fiber Mach-Zehnder interferometer (free spectral range (FSR) = 5.871 ± 0.004 MHz, see the Supplementary Information). M, Million. **b** UHQR ring-down time (*τ*) measurement at 1570 nm with calculated loaded and intrinsic *Q* factors (*Q*_L_ and *Q*_in_). The experimental data (blue curve) is fitted with an exponential curve (orange curve). **c** Summary of the total (blue circle) and intrinsic (blue dot) linewidths for the control and total (orange circle) and intrinsic (orange dot) linewidths for the UHQR devices from 1550 nm to 1600 nm. **d** Measured intrinsic *Q* from 1550 nm to 1600 nm for the control (blue dot) and UHQR (orange dot) resonators. **e** Photo-thermal absorption loss measurement from 1550 nm to 1600 nm for UHQR and control. The highest UHQR 422 Million intrinsic *Q* at 1570 nm and the absorption-limited *Q* (*Q*_abs_) of 3.4 Billion at 1600 nm are indicated. The non-absorption loss is fit to the surface scattering loss model (dash lines). The blue dot, cross, and diamond data represent the intrinsic, absorption, and non-absorption losses for the control resonators. The orange dot, cross, and diamond data represent the intrinsic, absorption, and non-absorption losses for the UHQR resonators.

The Q is measured using both the radio frequency (RF) calibrated fiber Mach-Zehnder interferometer (MZI) and ring-down techniques^2,15,27,41^, described in further detail in the Supplementary Information. Figure 2a shows a multiple free spectral range (FSR) transmission scan of the UHQR using a fast-tunable laser (see the “Methods” section), and an FSR of 2.713 GHz is measured. Resonance splitting is observed in some of the resonances (shown in the insets) due to a combination of coupling between the clockwise (CW) and counterclockwise (CCW) propagating modes and the very narrow resonator linewidth. Such splitting has been previously observed in ultra-high Q whispering-gallery-mode or microtoroid resonators^42–45^. The insets in Fig. 2a reveal individual resonance modes and random variation in resonance splitting across different modes.

**Fig. 3:**
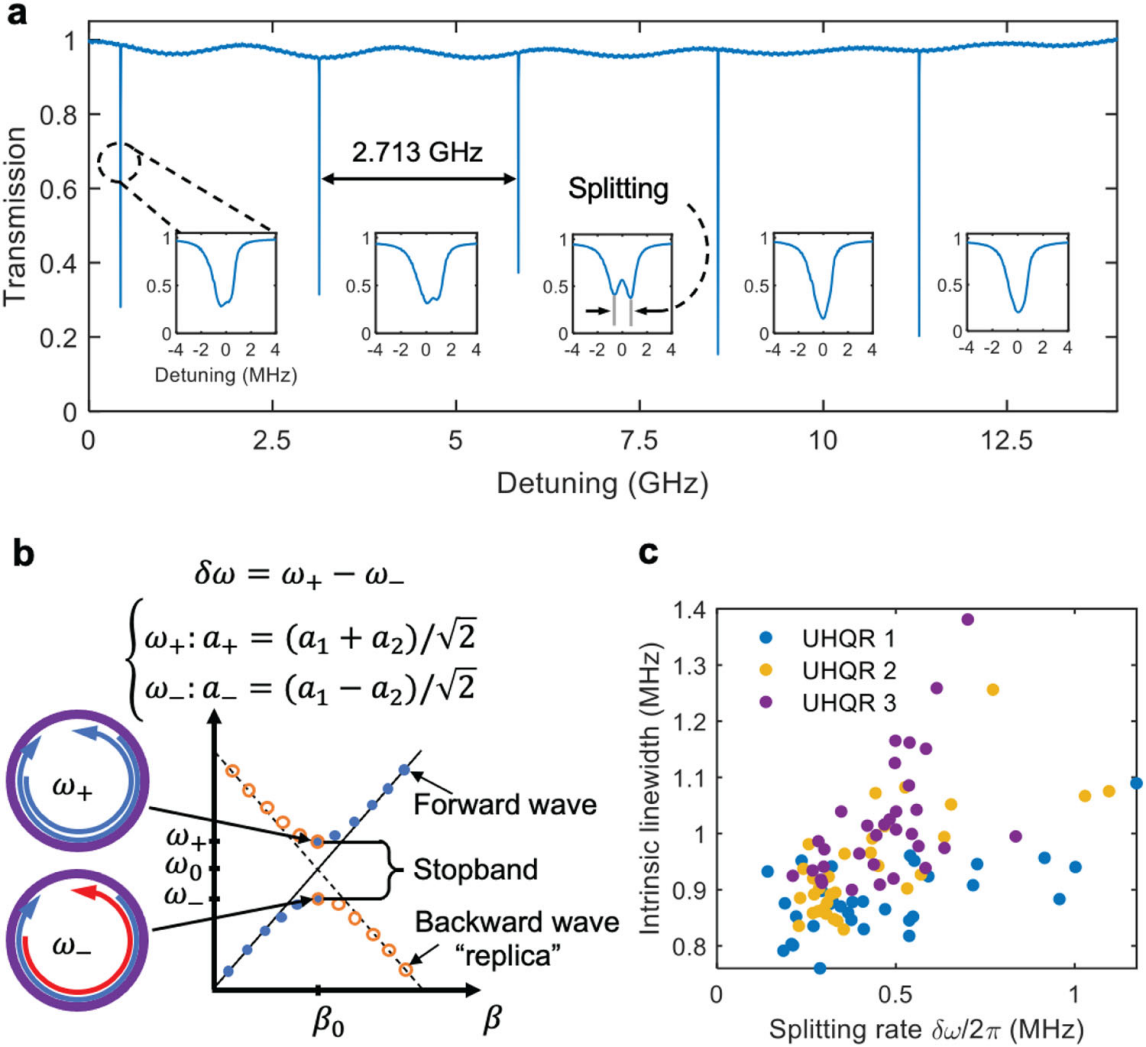
Resonance splitting. **a** Spectral scan across five free spectral ranges (FSRs) around 1550 nm confirms single transverse electrical field (TE) mode operation, blue curve. Details of individual resonances reveal random splitting. **b** Resonance splitting via coupling between forward and backward propagating waves. **c** Scatter plot of the measured intrinsic linewidth versus the splitting rate near 1550 nm for three ultra-high Q resonator (UHQR) devices fabricated on the same wafer. Blue dot, yellow dot, and purple dot represent UHQR1, UHQR2, and UHQR3 devices, respectively.

We use the coupled-mode equation (CME) method to model resonance splitting by incorporating the mode coupling^37,38,46,47^ as described in the Supplementary Information. Splitting can be intentionally achieved by adding Bragg gratings^48,49^ or by placing scatterers near the resonator waveguide^44,50,51^. The most likely cause of splitting here is random defects in the waveguide core due to surface and bulk defects^52^ and roughness induced random backscatter^53^. Random splitting variation can occur as a function of the number of defects on the waveguide surface, the relative distance between the defect particles, and the position of the particles on the waveguide surface^44,50,51^ (see Fig. 1d). For a single split resonance, the coupling between the CW and CCW modes creates a stopband resulting in resonance splitting (illustrated in Fig. 3b). In the presence of surface roughness, the splitting and the scattering loss of the waveguide both increase with roughness^38^ and across different wafers with different fabrication processes, splitting and loss can exhibit a strong correlation^39^. In Fig. 3c we plot the intrinsic linewidth versus the splitting rate for 96 measured resonances near 1550 nm for three different devices fabricated on one wafer. Positive correlation between linewidth and splitting rate is observed with a linear fit slope of 0.11, 0.48, and 1.55, yielding a linear correlation of 0.82, 0.73, and 0.76, respectively. These results indicate that splitting randomness is due to random defect particles and waveguide sidewall roughness, while at the same time correlating with loss. Moreover, the resonances with less evident splitting also exhibit larger extinction ratios at resonance. This implies a narrower linewidth given that the resonator is under-coupled^54^. Random resonance splitting is generally undesirable, leading for example to multimode or unstable lasing in Brillouin lasers^2,55^, or the disturbance of phase-matching conditions in Kerr optical frequency combs^5^.

To measure the resonator Q we employ two different techniques, RF calibrated MZI and time-resolved optical ringdown^2,27^. Further details of these two techniques are given in the Supplementary Information and the calibration details are described in the Methods section. The MZI FSR is calibrated to 5.871 MHz with a confidence interval of 0.004 MHz, corresponding to a relative error of 0.7%. This error propagates into the linewidth, *Q*, and FSR measurements. The measured transmission resonance shape is fit to a Lorentzian profile and the total linewidth *γ*_*T*_, loaded *Q* factor *Q*_*0*_ = *ω*_*0*_*/γ*_*T*_, coupling rate *γ*_ex_, intrinsic linewidth *γ*_in_, and intrinsic *Q* factor *Q*_in_ = *ω*_*0*_*/γ*_in_, and waveguide loss *α*, are extracted from this fit. Measurements for the non-split resonances, from 1550 to 1600 nm, for the UHQR and control devices are plotted in Fig. 2c–e. The highest intrinsic *Q* of 422 ± 11 Million is measured at 1570 nm for the UHQR. The spectral plots of the total linewidth, intrinsic linewidth, and coupling rate are given in the Supplementary Information. The 422 Million Q Lorentzian fit at 1570 nm results in a relative confidence interval of 2.4% at that wavelength, leading to a confidence interval of $\sqrt {\left( {0.7{\mathrm{\% }}} \right)^2 + \left( {2.4{\mathrm{\% }}} \right)^2} = 2.5{\mathrm{\% }}$ for the *Q* and linewidth measurement. The FSR and finesse measurements have a relative error of 0.7% from the MZI calibration. From these measurements, we calculate the total and intrinsic resonance linewidths at 1570 nm to be 906 ± 23 kHz and 453 ± 11 kHz respectively. The FSR at 1570 nm is 2.720 ± 0.019 GHz as shown in Supplementary Fig. 5 with a corresponding 3005 ± 21 finesse. To confirm the RF calibrated MZI UHQR *Q* measurements, we perform ring-down measurements (see “Methods” and Supplementary Information for further details), resulting in a loaded *Q* of 217 ± 4 Million. The spectral scan at 1570 nm suggests this resonance is at critical coupling, and the intrinsic *Q* is therefore 434 ± 9 Million. This confidence interval comes from the fitting of the ring-down. The spectral and temporal intrinsic *Q* measurements at 1570 nm are in strong agreement, given their overlapping confidence intervals.

### Loss reduction

The UHQR shows a significant loss reduction compared to the control resonator. To quantify the absorption and scattering loss contributions, we measure the photothermal absorption loss bistable response (see “Methods” and Supplementary Information for more detail)^33,38,39,56^. The photothermal effect induces a resonance redshift comparable to or larger than the resonance linewidth, a result of the on-chip absorption heating, shown in Supplementary Fig. 6. Coupling our model of the heat transfer in the resonator with the measured redshift and on-chip power, we quantify the absorption loss (see Supplementary Information). We use a ~1 ms sweep rate across the resonance as the modeled thermal time constant is ~0.1 ms^57^. Hydrogen impurity related absorption has been identified as a loss contribution in the ultra-low loss regime, with an absorption peak near 1520 nm^30,33,39,56^.

The measured absorption and calculated scattering losses as a function of wavelength are shown in Fig. 2e. The narrowest absorption-limited linewidth is 51 kHz at 1600 nm, corresponding to an absorption-limited Q of 3.4 Billion. For further details on the loss measurement technique see the Methods and Supplementary Information. This result indicates the potential for quality factors in excess of 1 Billion if scattering losses are reduced (to values less than those due to absorption) or eliminated. In order to assess the potential impact of the change of the optical mode due to the blanket ~5 nm nitride layer, we modeled the mode profile and confinement factor (see Supplementary Information) and confirm that there is a negligible change due to waveguide geometry. Table 1 shows a breakdown of the measured total and absorption loss and calculated scattering loss for the UHQR and control samples. We observe an order of magnitude decrease in scattering loss and a twofold decrease in absorption loss due to the ~5 nm blanket nitride layer and extra anneal step.

**Table 1:** Contribution from different loss origins.

Losses at 1570 nm	UHQR	Control
Scattering loss (dB m^−1^)	0.046	0.143
Absorption loss (dB m^−1^)	0.014	0.031
Total loss (dB m^−1^)	0.060	0.175

Scattering and absorption loss contributions. Measured total and absorption loss and calculated scattering loss for the ultra-high Q resonator (UHQR) and control devices indicating an order of magnitude decrease in scattering loss and a twofold decrease in absorption.

We fit the measured non-absorption loss to a scattering model as described in the Supplementary Information. Assuming a high-aspect-ratio waveguide design with a root mean square (RMS) sidewall roughness of 1 nm and a correlation length of 50 nm, a scattering loss on the order of 0.001 dB m^−1^ is calculated. Scattering losses on the order of 0.1 dB m^−1^ are calculated for the top and bottom surfaces of the waveguide, assuming an RMS roughness of 0.2 nm and a correlation length of 10 nm. Using these results we estimate the effective waveguide top surface roughness to be 0.21 and 0.32 nm for the UHQR and control devices, respectively, using a correlation length of 10 nm^30,31,39^. In addition, an increase in waveguide thickness will increase the mode confinement and consequently increase the scattering loss if the surface roughness stays approximately constant. However, we observe a decrease in loss, supporting our conclusion that the UHQR has lower surface roughness.

The increase in absorption loss at shorter wavelengths (Fig. 2e) suggests a likely hydrogen-impurity-related absorption peak near 1520 nm. To profile the hydrogen impurity concentration, SIMS is performed on sample wafers with both non-etched UHQR waveguide design and control waveguide design. The results, shown in Table 2, show that the hydrogen concentration was reduced by one order of magnitude on the sample with ~5 nm blanket nitride layer and subsequent 30-minute anneal. This supports our observation of absorption loss reduction as shown in Fig. 2e. Another component of surface absorption is due to surface reconstruction of dangling bonds^38,40^. Hydrogen at these interfaces can be attached to reconstructed defect bonds such as Si–H, N–H, and Si–O–H, as illustrated in Fig. 1e.

**Table 2:** Hydrogen concentration profile.

Atoms per cm^3^	UHQR	Control
Top of ~5 nm blanket nitride layer	1.0 × 10^21^ (1.11%)	NA
Top of LPCVD deposited waveguide core	5.7 × 10^20^ (0.63%)^a^	6.6 × 10^21^ (7.33%)
Bottom of LPCVD deposited waveguide core	2.7 × 10^20^ (0.30%)	1.6 × 10^21^ (1.78%)

Secondary ion mass spectroscopy (SIMS) is used to compare an ultra-high Q resonator (UHQR) sample wafer with a low-pressure chemical vapor deposition (LPCVD) core, a ~5 nm blanket silicon nitride layer, and a 30-minute anneal, to a control sample wafer with only an LPCVD nitride core.

^a^Interface between LPCVD deposited waveguide core and ~5 nm blanket nitride layer.

## Discussion

In this paper, we report a significant advancement in photonic integrated all-waveguide resonator performance and ultra-low loss waveguides, demonstrating a 422 Million intrinsic Q, a 453 kHz intrinsic linewidth, a 906 kHz loaded linewidth, and a corresponding linear loss of 0.060 dB m^−1^. These results represent, to the best of our knowledge, the highest Q and lowest linewidth demonstrated to date for an all-waveguide resonator as well as the lowest waveguide loss achieved with a silicon nitride waveguide with a deposited SiO_2_ upper cladding. The resonator supports only a single TE mode by designing high bending loss for the TM mode. This single polarization mode operation results in a single FSR and a more robust design as compared to dual-mode resonators^27^. The large mode volume and area (~2.9 × 10^6^ μm^3^ and 39.6 μm^2^) support large photon numbers, an important aspect of low fundamental laser linewidth lasers^2,58–60^. Ultra-high Q cavities, such as the one reported in this paper, in combination with feedback techniques such as Pound-Drever-Hall (PDH)^61,62^, can significantly narrow the laser integral linewidth when used as a reference cavity^63^. The 3.4 Billion absorption-loss-limited Q and corresponding 57 kHz absorption-loss-limited linewidth, are measured using a photothermal measurement technique. These measurements indicate the potential for photonic integrated waveguide resonators to exceed 1 Billion Q if scattering losses are reduced to below the current absorption losses. Error analysis is important to determine the Q, linewidth, and finesse with confidence for devices with this level of performance. The MZI spectral Q and linewidth measurements have a 2.5% confidence interval while the ringdown measurements result in a 2.1% confidence interval. We calculate a 0.7% relative error for FSR and finesse measurements. These spectral and temporal measurements are in a strong agreement given their overlapping confidence intervals. As waveguide resonator technology continues to improve, sophisticated measurement techniques will also need to improve in resolution and accuracy.

This level of performance is achieved through the introduction of a thin (~5 nm) blanket nitride layer with our prior high-aspect-ratio design^26,29^ that covers both the LPCVD deposited core and thermal oxide cladding, followed by a subsequent anneal prior to TEOS-PECVD upper oxide cladding deposition. Our measurements provide strong evidence that the blanket nitride layer reduces sidewall and top-side nitride scattering losses by a factor of 10 and lowers absorption loss by a factor of 3 over the low loss control sample. SIMS measurements on unetched UHQR and control silicon nitride samples reveal an order of magnitude decrease in the UHQR design in hydrogen concentration at the top of the LPCVD 40 nm nitride. The decrease in absorption losses further suggests that the combination of the thin nitride layer and subsequent anneal lower the absorption losses due to hydrogen impurities and uncompensated dangling bonds at the LPCVD deposited top nitride surface. The sub-MHz resonance linewidth allows us to resolve resonance splitting not observed in our previous lower Q designs. We apply a CME mode-coupling model that allows us to measure and further analyze underlying loss mechanisms and come up with strategies to further increase Q and lower loss. The linear fit slope between linewidth and splitting rate indicates that splitting randomness is due to random defect particles and waveguide sidewall roughness, which correlate linearly with scattering losses. We identify the most likely cause of splitting to be random defects in the nitride waveguide core due to surface and bulk defects and roughness induced random backscatter. Since the random splitting can occur as a function of the number of the defects on the waveguide surface, the relative distance between the defect particles, and the position of the particles on the waveguide surface, we believe that improvements in nitride deposition and post processing can further reduce losses as well as alternative waveguide processing techniques and geometries that produce smoother surfaces^64^. From the SIMS results, we suspect that the hydrogen-impurity has two origins, which are dangling bonds at the nitride surface after LPCVD deposition, and moisture from exposure to air. In the future, measurements of the individual losses with each improvement (i.e., after blanket nitride layer, after annealing, after the wide waveguide etch) will be performed to further improve our understanding of the loss mechanisms. Mitigation strategies include additional targeted annealing steps and dangling bond passivation to prevent the formation of unwanted molecules. It may also be possible to further increase the Q by utilizing a TM mode, which is less susceptible to waveguide roughness and has lower propagation losses^27,28^.

In addition to frequency noise reduction and linewidth narrowing, all-waveguide integrated cavities have the potential to play a role in laser stabilization. The degree of stability is relative to the particular application and the required integration intervals, for example, the length of time before an optical atomic clock laser drifts out of the locking range of an atomic resonance linewidth relative to the open-loop clock cycle^8^ or the integral laser linewidth that is measured by carrier jitter and the Allan deviation^65^ and close-to-carrier noise^63^. Frequency stabilization can be determined by the resonator’s close-to-carrier frequency noise, jitter, and long-term drift. Stabilization of the low-frequency noise components (e.g., 1 Hz and less) will require cavity designs that also have near-zero thermal expansion coefficients^66–68^ and dampened responses to the environmental disturbances^69^ determined in large part by packaging and mounting as well as the resonator material and design. These aspects of resonator stabilization are the subject of ongoing research for waveguide resonators. The results presented in this paper demonstrate promise to bring the performance of bulk optic and etched resonators to planar all-waveguide solutions and pave the path towards integrated Billion Q all-waveguide cavities for atomic clocks, nonlinear optics, quantum computing, and communications, precision spectroscopy and energy-efficient coherent communications systems.

## Methods

### Fabrication process

The substrate and lower cladding consist of a 15-µm-thick thermal oxide grown on a 100-mm diameter silicon wafer. The main waveguide layer is a 40-nm-thick stoichiometric Si_3_N_4_ film deposited on the lower cladding thermal oxide using LPCVD. A standard deep ultraviolet (DUV) photoresist layer was spun and then patterned using a DUV stepper. The high-aspect-ratio waveguide core is formed by anisotropically dry etching the Si_3_N_4_ film in an inductively coupled plasma etcher using a CHF_3_/CF_4_/O_2_ chemistry. Following the etch, the wafer is cleaned using a standard Radio Corporation of America (RCA) cleaning process. For the UHQR, an additional thin layer of silicon nitride was deposited followed by a 30-min anneal at 1100 °C in an oxygen atmosphere, without additional etching. A 6-µm-thick silicon dioxide upper cladding layer was deposited in two 3-µm steps using plasma-enhanced chemical vapor deposition (PECVD) with tetraethoxysilane (TEOS) as a precursor, followed by a final two-step anneal at 1050 °C for 7 h and 1150 °C for 2 h. The fabrication process flow is illustrated in Supplementary Fig. 1.

### Q and loss measurements

We used the TLB-6730 Velocity™ Widely Tunable Laser in these measurements. A ~40 m unbalanced fiber-based RF calibrated MZI was used resonator to measure the Q. To calibrate the MZI FSR, a radiofrequency (RF) electro-optic phase modulator (EOM) was used to create two sidebands. While scanning across a resonance, the two sidebands are used to calibrate the MZI FSR. Three calibrations were performed with three different RF frequencies, 10, 20, and 30 MHz. The MZI FSR is measured to be 5.871 ± 0.004 MHz. We simultaneously scan the laser through both the MZI and the UHQR and control devices producing the calibrated scans reported. A standard cavity ring-down technique was used to confirm the RF calibrated MZI measurements. For this measurement, a fiber laser was frequency swept around a resonator resonance by applying a triangular signal to the laser piezoelectric frequency control. Meanwhile, a 10 kHz square wave was applied to a 10 GHz intensity modulator to serve as the input laser power gate (on-off). A Thorlabs PDB-450C with a 150 MHz bandwidth was used to monitor the optical signal. The laser frequency is ramped slowly enough to allow charge and discharge of the resonator. The ringdown decay time (*τ*) is measured by fitting the measured exponential decay. The loaded Q factor is evaluated using *Q*_*L*_ = *ωτ* and the total loss rate is calculated as *γ*_*T*_ = *1/τ*. From the spectral linewidth and RF calibrated MZI Q measurements, the resonance extinction ratio indicates the ratio between the intrinsic and coupling losses. Given that these resonator devices are under-coupled (see the Supplementary Information and Supplementary Fig. 4 for details), we are able to calculate the intrinsic loss from the ringdown time.

### SIMS measurements

SIMS was performed on two wafer samples each with LPCVD deposited 40 nm silicon nitride thin film on the thermal oxide. One wafer has the ~5 nm blanket nitride layer followed by a 30 min anneal. The other wafer is 40 nm LPCVD core only without the blanket nitride layer and subsequent anneal. The wafer samples were then sent to EAG Laboratories for SIMS to profile the hydrogen impurity concentration.

## Supplementary information

Supplementary Information

## Data Availability

The data that support the plots within this paper and other findings of this study are available from the corresponding author on reasonable request.
