# Supplementary material for: 422 Million intrinsic quality factor planar integrated all-waveguide resonator with sub-MHz linewidth
Source: Nat Commun. 2021 Feb 10;12:934. doi: 10.1038/s41467-021-21205-4 (PMC7876138; doi:10.1038/s41467-021-21205-4)
Supplement: Supplementary file 1 — Supplementary Information [file 41467_2021_21205_MOESM1_ESM.pdf]

SUPPLEMENTARY INFORMATION

422 Million Intrinsic Quality Factor Planar Integrated All-Waveguide Resonator with Sub-MHz Linewidth

Matthew W. Puckett[1], Kaikai Liu[2], Nitesh Chauhan[2], Qiancheng Zhao[2], Naijun Jin[3], Haotian Cheng[3], Jianfeng Wu[1], Ryan O. Behunin[4], Peter T. Rakich[3], Karl D. Nelson[1], Daniel J. Blumenthal[2*]
[1]Honeywell International, Phoenix, AZ, USA.
[2]Department of Electrical and Computer Engineering, University of California Santa Barbara, Santa Barbara, CA, USA.
[3]Department of Applied Physics, Yale University, New Haven, CT, USA.
[4]Department of Physics and Astronomy, Northern Arizona University, Flagstaff, AZ, USA.
These authors contributed equally: Matthew W. Puckett, Kaikai Liu.

*Corresponding author (danb@ucsb.edu)

Supplementary Note 1: Introduction

In this Supplementary Information, we reveal more details on the device fabrication processes; we discuss in detail the single mode operation and the bus-to-ring coupling design in our resonators through a simulation study combined with experimental data; we provide a detailed description of the spectral scan experiments for linewidth measurements and the ringdown measurements; we describe our theoretical modeling of the scattering loss and resonance splitting; we reveal in detail the principles and methods of how we quantify the absorption loss from the photothermal effect observed in the spectral scan of the resonance.

Supplementary Note 2: Fabrication flow and resonator design

Supplementary Fig. 1 shows the fabrication process flow with the redeposition-and-anneal steps indicated as step 3 and 4, where we deposit a thin layer of silicon nitride and carry out subsequent annealing at 1100 °C for 30 minutes. This step yields an additional ~5 nm silicon nitride layer. The ultra-high Q resonator (UHQR) devices were fabricated with step 3 and 4 while the control resonators were fabricated without step 3 and 4.

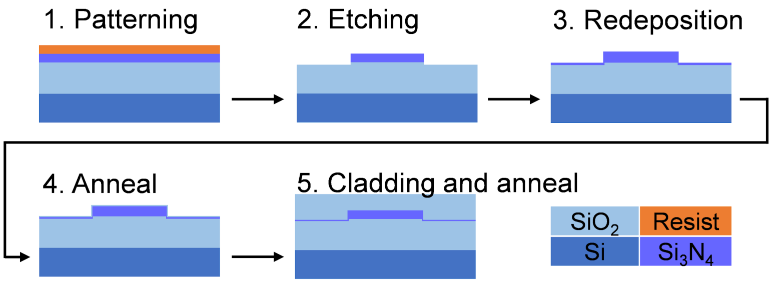

Supplementary Fig. 1. Fabrication process flow. Step 1: Silicon nitride core patterning. Step 2: Silicon nitride core etch. Step 3: Nitride redeposition. Step 4: Anneal. Step 5: Upper oxide cladding and anneal.

Using the measured refractive indices of the core and cladding materials that can be found in the Supplementary of our previous work[1], we perform mode simulations with Lumerical to calculate the effective indices and bending losses, as summarized in Supplementary Fig. 2, which indicates that both our bus and resonator waveguides only support the fundamental TE mode. In the bending radius range shown in Supplementary Fig. 2**f**, the fundamental TM mode is not found in the mode solver and the fundamental TE mode has a critical bending radius of ~6.8 mm.

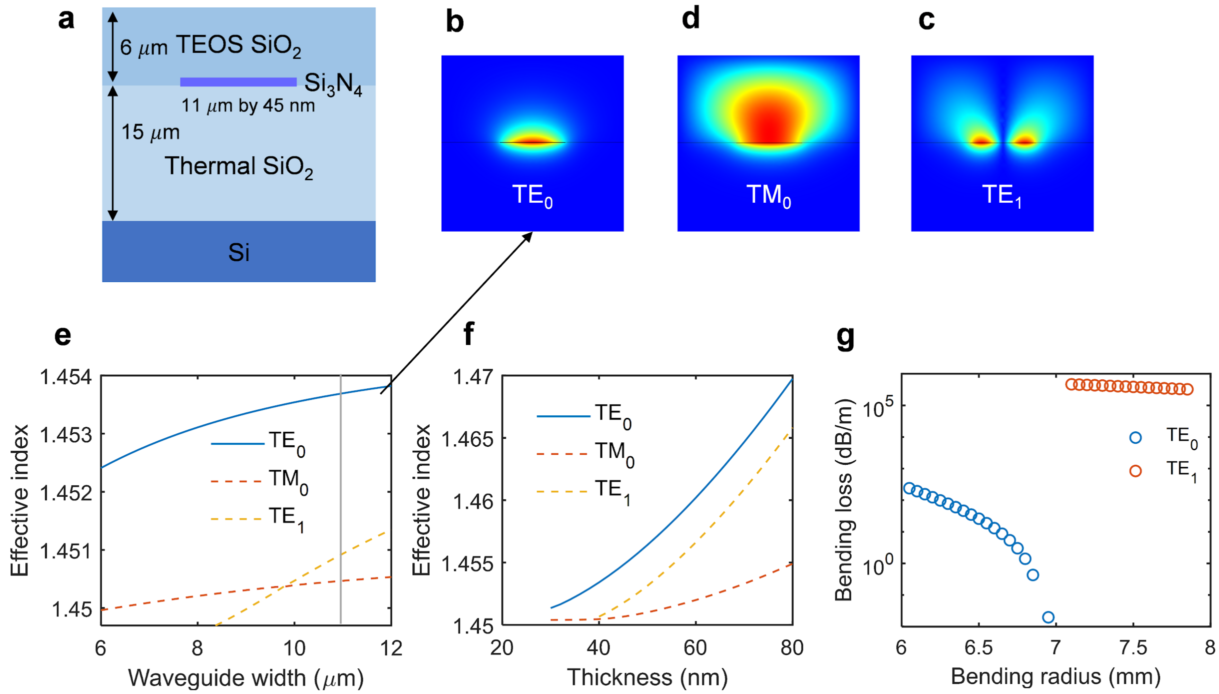

Supplementary Fig. 2. Single mode operation. a The resonator waveguide geometry. **b c d** Mode profiles of the fundamental TE mode, unsupported TM_0 mode, and unsupported TE_0 mode for the waveguide geometry shown in **a**. **e** Effective index versus waveguide width with the waveguide thickness fixed at 45 nm. **f** Effective index versus waveguide thickness with the waveguide width fixed at 11 μm. **g** Simulated bending loss for the modes TE_0 and TE_1 shows that a bending radius larger than 7 mm only supports the TE_0 mode. Within this bending radius range shown here the TM mode does not exist due to the significantly higher TM bending loss.

Since the Lorentzian fit used to extract the intrinsic and coupling loss rates of the resonances does not distinguish one from another, we perform a Comsol simulation and a numerical calculation to fit the extracted coupling loss rate and to distinguish the intrinsic loss from the coupling loss[8]. Supplementary Fig. 3 summarizes the simulation and fitting for coupling loss from 1550 nm to 1600 nm, and Supplementary Fig. 3**c** shows good agreement between the coupling simulation and the direct measurement of a test structure. The test structure is on a stage, the temperature of which is stabilized at the level of 1 mK variance. Changing the temperature of the stage is not observed to have any appreciable effect on the coupling. The coupler is weakly tapered to avoid any excess

loss, as shown in a microscope image of the device in Supplementary Fig. 3**b**. Should there be excess loss γ besides the coupling coefficient κ_c at the coupler, the resonator total linewidth would be expressed as,

$$\gamma_T = \frac{c}{n_g L}(\alpha L + \kappa_c + \gamma). \quad (1)$$

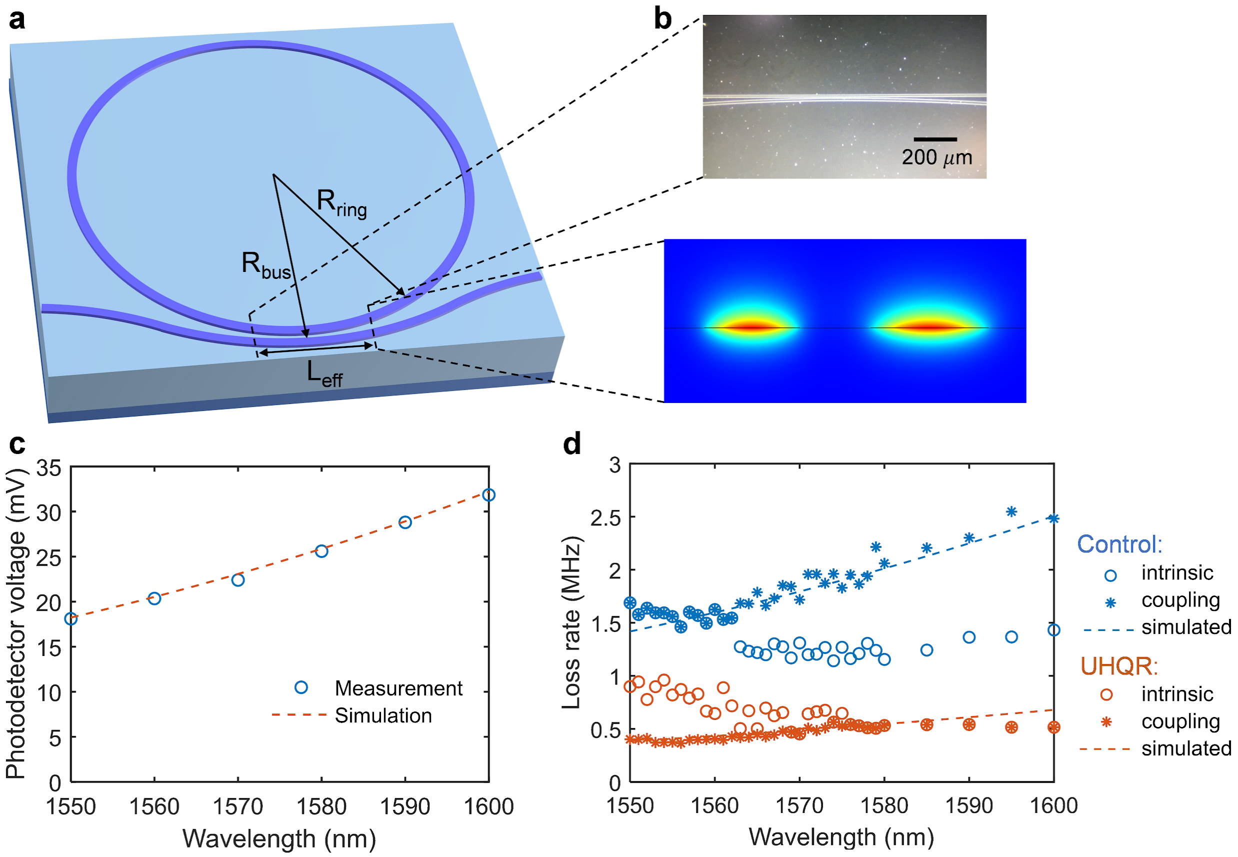

Supplementary Fig. 3. Weakly tapered coupling design, coupling simulation and measurement. a Weakly tapered coupling design. **b** Microscope image of the coupler region and simulated mode profile for the coupler cross section waveguides. **c** Direct measurement of the coupling output on a photodetector fitted by a simulation curve with an arbitrary scaling coefficient. **d** Coupling rates for both the UHQR and control devices fitted with simulation curves as shown by the dash lines.

Supplementary Note 3: MZI calibration and ringdown measurement

To calibrate the fibre Mach-Zehnder interferometer (MZI) free spectrum range (FSR), we employ an electro-optic modulator (EOM) to add two sidebands the distance between which is twice the modulation RF frequency to serve as the frequency detuning reference, as illustrated in Supplementary Fig. 4**a**. We carried out three calibrations with three RF frequencies, 10, 20, and 30 MHz. The FSR is calibrated to be 5.871±0.004 MHz. By sweeping the laser frequency, feeding the laser power into both a resonator device and the MZI, and monitoring the two optical signals simultaneously, the sidebands in the resonator transmission provide the frequency reference, and counting the MZI fringes measures the FSR. To further confirm values produced by the MZI

measurement, a ringdown experiment is carried out at 1550 nm for both the UHQR and control devices, and the ringdown results agree well with the linewidth measurements, as shown in Supplementary Fig. 4.

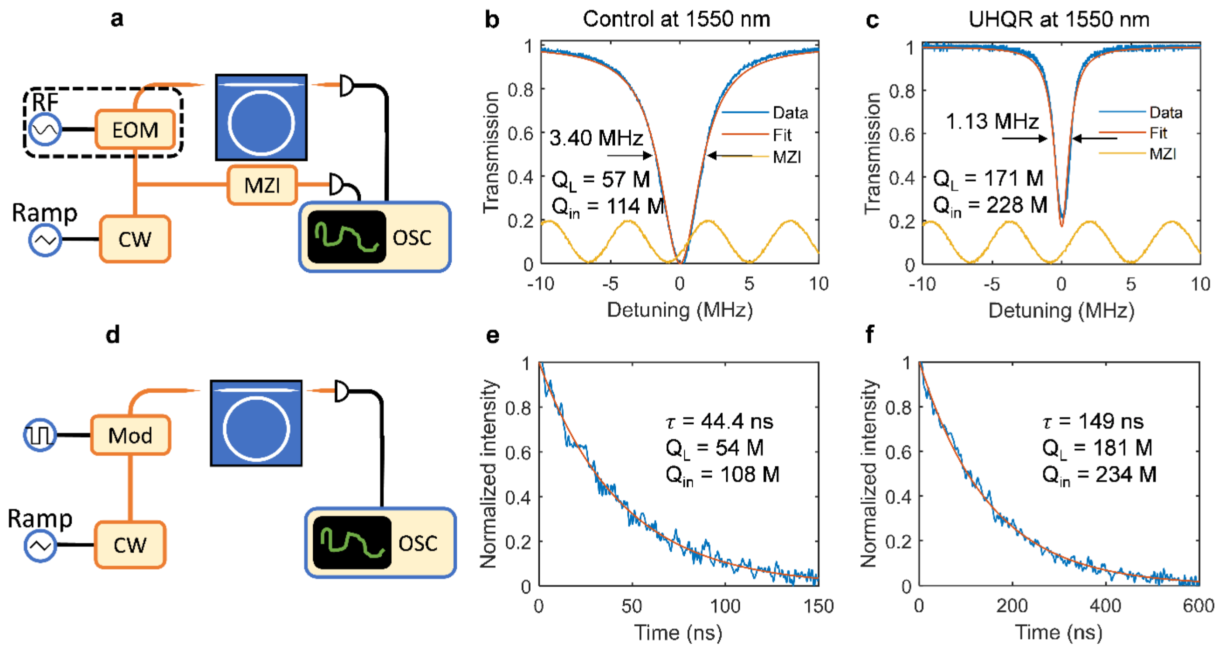

Supplementary Fig. 4. MZI calibration, RF calibrated MZI linewidth measurement, and ringdown experiment at 1550 nm. a Experiment setup diagram for MZI calibration and linewidth measurement. The EOM was used only to create two sidebands when calibrating the MZI FSR and was not used during the spectral linewidth measurements. **d** Diagram for ringdown experiments. A ramp signal sweeps the frequency of a CW laser and a square wave is applied onto the intensity modulator serving as a switch between optical power "on" and "off". **b c** RF calibrated MZI linewidth measurement at 1550 nm for both the UHQR and control devices. **e f** Ringdown experiment at 1550 nm for both the UHQR and control devices.

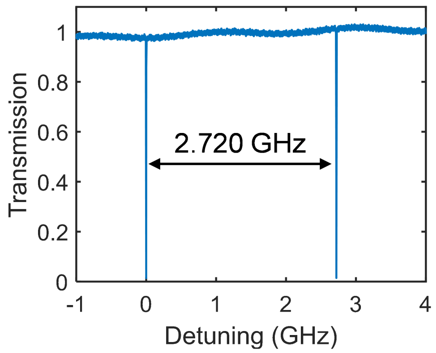

Supplementary Fig. 5. FSR at 1570 nm is measured to be 2.720 GHz using the RF calibrated MZI.

Supplementary Note 4: Coupled mode equation for resonance splitting.

To describe the mode coupling between the clockwise (CW) and counterclockwise (CCW) modes and the consequent resonance splitting, the coupled mode equation (CME) method is widely used as follows,

$$\frac{da_1}{dt} = -\left(i\Delta\omega + \frac{\gamma_T}{2}\right)a_1 + i\frac{g}{2}a_2 + i\sqrt{\gamma_{ex}}s_{in},$$

$$\frac{da_2}{dt} = -\left(i\Delta\omega + \frac{\gamma_T}{2}\right)a_2 + i\frac{g}{2}a_1,$$

$$s_{out} = s_{in} + i\sqrt{\gamma_{ex}}a_1, \qquad (2)$$

where a_1 and a_2 denote the CW and CCW mode amplitudes, s_{in} is the input mode, $\gamma_T = \gamma_{in} + \gamma_{ex}$ is the total loss including the intrinsic loss γ_{in} and the external coupling loss γ_{ex}, the mode coupling coefficient is a complex number $g = g_R + ig_I$ and $\Delta\omega$ is scan detuning. Solving the CME yields the doublet transmission lineshape where the mode coupling g, the splitting rate $\delta\omega$ and the linewidth difference $\delta\gamma$,

$$\left|\frac{s_{out}}{s_{in}}\right|^2 = \left|1 - \frac{\gamma_{ex}/2}{i(\Delta\omega - g_R) + (\gamma_T - g_I)/2} - \frac{\gamma_{ex}/2}{i(\Delta\omega + g_R) + (\gamma_T + g_I)/2}\right|, \qquad (3)$$

$$\delta\omega = 2g_R, \delta\gamma = 2g_I. \qquad (4)$$

Equation (3) and (4) are employed to fit the split resonances with g_I is set to be 0 to extract the intrinsic linewidth $\gamma_T = \gamma_{in} + \gamma_{ex}$, and the splitting rate $\delta\omega$.

Supplementary Note 5: Scattering loss and mode coupling modeling

The widely-used model to estimate waveguide scattering loss is the fully three dimensional volume current method (3D-VCM)[2–4], both scattering loss γ_s and mode coupling $g = g_R + ig_I$ can be estimated from the waveguide roughness profile. The far-field electric field produced by the roughness induced volume current and consequent coupling rate between the guide mode and radiation continuum are expressed as follows[4],

$$\mathbf{S}_m(\theta, \phi) = \sqrt{\frac{\omega^3}{16\pi^2\epsilon_0 c^3 U_m}} \int \Delta\epsilon(\mathbf{r})\mathbf{E}_m(\mathbf{r}) \cdot \left(1 - \hat{\mathbf{k}}\hat{\mathbf{k}}\right)e^{-ik_0\hat{\mathbf{k}}\cdot\mathbf{r}}d^3\mathbf{r}, \qquad (5)$$

$$\Gamma_{m,m'} = \omega \int \mathbf{S}_m^*(\theta, \phi) \cdot \mathbf{S}_{m'}(\theta, \phi) \sin\theta \, d\theta d\phi, \qquad (6)$$

where m denotes the CW and CCW modes, U_m is the mode energy in the waveguide, and $\Delta\epsilon(r)$ includes the roughness information. We can find that $\gamma_s = \Gamma_{11}$ and $g_I = \Gamma_{12}$. With the first order perturbation theory, the coupling rate between the CW and CCW modes can be expressed as,

$$g_R = \frac{\omega}{2U_m} \int \Delta\epsilon(\mathbf{r})\mathbf{E}_m^*(\mathbf{r}) \cdot \mathbf{E}_m(\mathbf{r})d^3\mathbf{r}. \qquad (7)$$

The integrations in Equation (5-7) incorporate the sidewall roughness,

$$R_{side}(u_z) = \langle f_{side}(z) f_{side}(z + u_z)\rangle = \sigma_{side}^2 \exp(-\frac{u_z}{L_{side}}), \qquad (8)$$

and the top surface roughness,

$$R_{top}(u_x, u_y) = \langle f_{top}(x,y) f_{top}(x+u_x, y+u_y)\rangle = \sigma_{top}^2 \exp(-\frac{u_x+u_y}{L_{top}}). \quad (9)$$

Our model for estimating the scattering loss and mode coupling is validated by our model estimate getting the same sidewall scattering loss value from the same calculation carried out in these references[3,5]. Here we generate a tentative estimation by choosing typical numbers for the waveguide roughness profiles: σ_{side}= 2 nm, L_{side} = 50 nm, σ_{top} = 0.3 nm, and L_{top} = 10 nm, which yields α_{top} = 0.101 dB m^{-1}, α_{side} = 0.001 dB m^{-1}, g_R = (2π) 0.632 MHz (from top roughness) + 0.001 MHz (from sidewall roughness) = (2π) 0.633 MHz. These estimates are of similar order of magnitude to the experimental measurements.

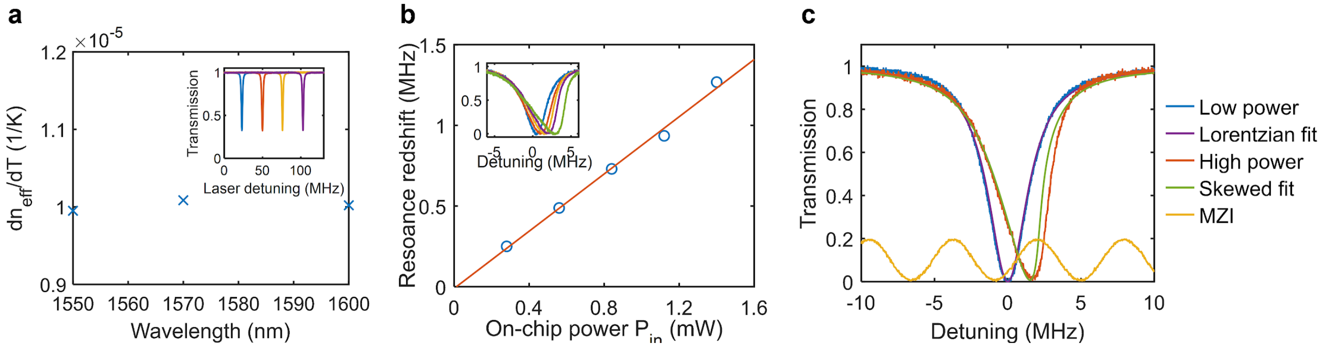

Supplementary Fig. 6. Photo-thermal heating and absorption loss measurement. a Effective index change with respect to temperature shows no wavelength dependence across the range from 1550 nm to 1600 nm. **b** Photothermal effect is amplified by higher on-chip power and the resonance redshift exhibits a linear relationship with the on-chip power. **c** Normal Lorentzian fitting for the lower power spectral sweeping and skewed Lorentzian fitting for the high-power spectral sweeping extracts the intrinsic loss and absorption loss rates.

Supplementary Note 6: Photothermal absorption loss measurement

On-resonance drop in transmitted power necessarily indicates power dissipation in a resonator: $P_{disp} = P_{in}(1 - T_{res})$. Part of the dissipated power is absorbed and converted into heat: $P_{abs} = \xi P_{disp}$, where ξ is the absorption loss fraction and the absorption loss rate can be expressed as $\gamma_{abs} = \xi \gamma_{in}$. Since only the waveguide is heated and the 1 mm thick Si substrate remains mostly undisturbed, the thermo-optic effect dominates, and thermal expansion is negligible. Using the thermo-optic coefficients of SiO_2 (0.95×10^{-5} K^{-1}) and SiN (2.45×10^{-5} K^{-1}) at 1550 nm reported in the literature[6,7], we perform a COMSOL simulation that simulates the thermal heating due to absorption heating and estimates the redshift given an absorption power: $\delta f_{res} = \alpha P_{abs}$, illustrated in the inset of Supplementary Fig. 6**b**. The simulation suggests R_{th}= 4.98 K W^{-1}, $\delta f_{res}/\delta T$= 1.23 GHz K^{-1}, and $\alpha = \delta f_{res}/P_{abs}$= 6.11 MHz mW^{-1}. To confirm the same thermo-optic coefficients at 1550 nm are valid for other wavelengths, we measure the resonance shift with a temperature increase and estimate the effective index change. Supplementary Fig. 6**a** shows that there is not an obvious wavelength dependence of the thermo-optic coefficients. A normal Lorentzian fit on the

lower power transmission lineshape extracts the linewidths. With the extracted linewidths as the input parameters, we perform another fitting on the high power skewed lineshape with the following equation,

$$T = 1 - \frac{\gamma_{in}\gamma_{ex}}{[\Delta\omega - 2\pi f_D(1-T)]^2 + (\gamma_{in}+\gamma_{ex})^2/4}, \tag{10}$$

where $f_D = \delta f_{res} / (1 - T) = \xi \alpha P_{in}$ is the only parameter to be extracted allowing ξ to be determined. Supplementary Fig. 6**c** demonstrates the fitting processes.

Supplementary References

1. Gundavarapu, S. *et al.* Sub-hertz fundamental linewidth photonic integrated Brillouin laser. *Nat. Photonics* **13**, (2018).
2. Barwicz, T. 3D Analysis of scattering losses due to sidewall roughness in microphotonic waveguides: High index-contrast. *Opt. InfoBase Conf. Pap.* **23**, 2719–2732 (2005).
3. Ciminelli, C., Dell'Olio, F., Passaro, V. M. N. & Armenise, M. N. Fully three-dimensional accurate modeling of scattering loss in optical waveguides. *Opt. Quantum Electron.* **41**, 285–298 (2009).
4. Li, Q., Eftekhar, A. A., Xia, Z. & Adibi, A. Unified approach to mode splitting and scattering loss in high-Q whispering-gallery-mode microresonators. *Phys. Rev. - At. Mol. Opt. Phys.* **88**, 1–11 (2013).
5. Lee, K. K. *et al.* Effect of size and roughness on light transmission in a Si/SiO_2 waveguide: Experiments and model. *Appl. Phys. Lett.* **77**, 1617–1619 (2000).
6. Arbabi, A. & Goddard, L. L. Measurements of the refractive indices and thermo-optic coefficients of Si_3N_4 and SiOx using microring resonances. *Opt. Lett.* **38**, 3878 (2013).
7. Trenti, A. *et al.* Thermo-optic coefficient and nonlinear refractive index of silicon oxynitride waveguides. *AIP Adv.* **8**, 025311 (2018).
8. Shah Hosseini, E., Yegnanarayanan, S., Atabaki, A. H., Soltani, M. & Adibi, A. Systematic design and fabrication of high-Q single-mode pulley-coupled planar silicon nitride microdisk resonators at visible wavelengths. *Opt. Express* **18**, 2127 (2010).
